# Acute genital ulcers in a young girl: a clinical challenge: Lipschütz ulcer^☆^

**DOI:** 10.1016/j.abd.2020.12.018

**Published:** 2022-07-13

**Authors:** Fabiola Schafer, Rodrigo Miranda

**Affiliations:** a Department of Medical Specialties, School of Medicine, Universidad de La Frontera, Temuco, Chile; b Department of Internal Medicine, School of Medicine, Universidad de La Frontera, Temuco, Chile

Dear Editor,

A healthy 9-year-old girl presented with an acute onset of painful vulvar ulcers and dysuria. Physical examination of genital mucosa showed well-defined deep ulcers with a fibrinous center and elevated red borders on the labia majora (Fig. 1). Ulcers were large over 1 centimeter in diameter, in a mirror pattern. She reported a high fever up to 38,5 °C, odynophagia, congestion, and malaise one week before. Serological tests were negative for herpes simplex virus, Epstein Barr virus, cytomegalovirus, human immunodeficiency virus (HIV), and venereal disease research laboratory (VDRL). Autoimmune tests were negative for antinuclear and anti-DNA antibodies. Complete blood tests and urine samples were normal. Hormone tests such as estradiol, prolactin, follicle-stimulating hormone (FSH), and luteinizing hormone (LH) were all normal for her age. The diagnosis of Lipschütz ulcer was made. The patient was started on topical anesthetic cream and the ulcers had total recovery after two weeks, with no scarring. She has not presented new episodes at one-year follow-up.

**Figure 1:**
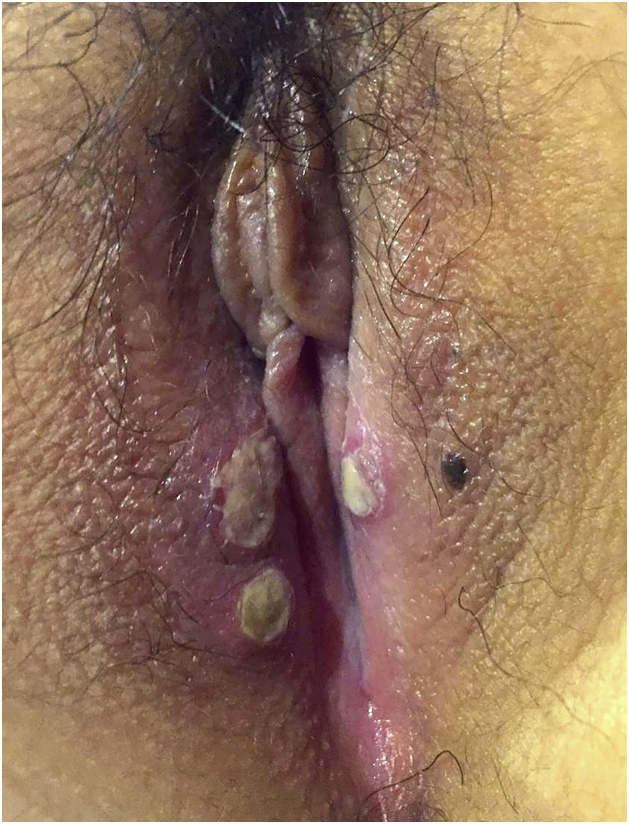
Well-defined deep ulcers with fibrinous center and elevated red borders on the labia majora. Ulcers are large over 1 centimeter of diameter, in a mirror pattern.

Lipschütz ulcer, also known as reactive non-sexually related acute genital ulcers, is a very uncommon clinical entity that typically occurs in sexually inactive young women.^1^ It is characterized by an abrupt onset, intense local pain, and dysuria. Its morphology is variable, often presenting as ‘kissing ulcers’ with a symmetric appearance on opposite sides of the vulva.^2^, ^3^ Also, necrotic ulcers with important oedema and erythema of the labia and inguinal lymphadenopathy have been described.^2^, ^4^ The ulcers can be single or multiple with raised, sharply demarcated borders. Most of them are often covered with gray exudate or a gray-black eschar.^3^ Typically, ulcers are located on the labia minora, but they can also be found on the labia majora, perineum, and in the lower vagina. It affects mainly adolescents and young women, and it is uncommon for children.^2^

Lipschütz ulcer is usually preceded by flu-like symptoms. Its etiology and pathogenesis are still unknown.^2^, ^3^ Although, some viruses or bacteria have been associated with this entity (Epstein-Barr virus, Mycoplasma and influenza A infection). The pathogenic mechanism is unclear, but a reactive process triggered by a distant infection with deposition of immune complex in the dermal vessels causing micro-thrombosis, and eventually leading to deep, necrotizing, painful ulcers is suspected.^4^ The diagnosis is made by exclusion, after ruling out other causes of genital ulcerations. Differential diagnosis includes ulcers of venereal and non-venereal origin, auto-immune diseases, trauma, and malignant tumors.^5^

The treatment is mainly symptomatic, with spontaneous resolution within 2‒6 weeks and without recurrences in most cases. Due to its self-limited evolution, local care is sufficient. Topical anesthetic, topical corticosteroids, and oral analgesics are usually indicated. On the other hand, if the patient has severe pain or malaise, hospitalization is indicated, so systemic steroids and broad-spectrum antibiotics are recommended.

Lipschütz ulcer is a challenge in clinical practice, it is usually under-diagnosed or misdiagnosed. Furthermore, there is high anxiety and confusion for patients and their families as the diagnosis of the herpes simplex virus is often presumptively made. Therefore, the authors highlight to keep in mind this unusual diagnosis especially in a young girl or adolescent with acute genital ulcers.

## Financial support

This article was funded by the 10.13039/501100005916Universidad de La Frontera [DI13-0051].

## Authors’ contributions

Fabiola Schafer: Approval of the final version of the manuscript.; drafting and editing of the manuscript; collection, analysis, and interpretation of data; participation in study design; critical review of the literature; critical review of the manuscript.

Rodrigo Miranda: Approval of the final version of the manuscript; drafting and editing of the manuscript; collection, analysis, and interpretation of data; participation in study design; critical review of the literature; critical review of the manuscript.

## Conflicts of interest

None declared.
